# Research priority setting for health policy and health systems strengthening in Nigeria: the policymakers and stakeholders perspective and involvement

**DOI:** 10.11604/pamj.2013.16.10.2318

**Published:** 2013-09-12

**Authors:** Chigozie Jesse Uneke, Abel Ebeh Ezeoha, Chinwendu Daniel Ndukwe, Patrick Gold Oyibo, Friday Onwe, Bhupinder Kaur Aulakh

**Affiliations:** 1 Department of Medical Microbiology/Parasitology, Faculty of clinical Medicine, Ebonyi State University, PMB 053 Abakaliki Nigeria; 2 Department of Community Medicine, Faculty of clinical Medicine, Delta State University, PMB 001 Abraka Nigeria; 3 Alliance for Health Policy and Systems Research (AHPSR) World Health Organization Avenue Appia 20 1211 Geneva 27 Switzerland

**Keywords:** Research, priority setting, health policy, health system strengthening, policymakers, Africa

## Abstract

**Introduction:**

Nigeria is one of the low and middle income countries (LMICs) facing severe resource constraint, making it impossible for adequate resources to be allocated to the health sector. Priority setting becomes imperative because it guides investments in health care, health research and respects resource constraints. The objective of this study was to enhance the knowledge and understanding of policymakers on research priority setting and to conduct a research priority setting exercise.

**Methods:**

A one-day evidence-to-policy research priority setting meeting was held. The meeting participants included senior and middle level policymakers and key decision makers/stakeholders in the health sector in Ebonyi State southeastern Nigeria. The priorities setting meeting involved a training session on priority setting process and conduction of priority setting exercise using the essential national health research (ENHR) approach. The focus was on the health systems building blocks (health workforce; health finance; leadership/governance; medical products/technology; service delivery; and health information/evidence).

**Results:**

Of the total of 92 policymakers invited 90(97.8%) attended the meeting. It was the consensus of the policymakers that research should focus on the challenges of optimal access to health products and technology; effective health service delivery and disease control under a national emergency situation; the shortfalls in the supply of professional personnel; and the issues of governance in the health sector management.

**Conclusion:**

Research priority setting exercise involving policymakers is an example of demand driven strategy in the health policymaking process capable of reversing inequities and strengthening the health systems in LMICs.

## Introduction

Developing countries worldwide are facing severe resource constraint, making it practically impossible for adequate resources to be allocated to key sectors of development including the health sector. According to Sabik and Lie [1], the scarcity of resources raises questions of justice and efficiency: how should limited health care resources be allocated? what health services should be publicly funded? How should indications for particular interventions be defined? Priority setting can provide answers to some of these questions. Priority setting (also known as resource rationing) has been defined as the distribution of limited resources among competing programs or people [2]. According to Lenaway and colleagues [3], it is a transparent, fair, legitimate and accountable process designed to guide decisions, a rational means to determine how resources are invested to address societal needs and to steer researchers towards topics of “national interest and priority”.

Priority setting is therefore required in every health care system because it guides investments in health care and health research and respects resource constraints.[4] Research priority setting for health policy is vital in order to channel resource allocation, as well as donor investment in health, to areas of highest priority; to address the issue of equity; and to reinforce the links between research, action and policy [5] Campbell [6] described priority setting as an important knowledge translation (KT) tool which identifies policy needs, research options and unites policy-makers and researchers before research begins.

It is pertinent to state however, that priority setting is a complex and one of the biggest challenges faced by all decision makers at all levels of all health systems, including macro (e.g. governments), meso (e.g. regional health authorities, hospitals), and micro (e.g. clinical programs) levels worldwide [7]. The demand for health services continues to outstrip the resources available to finance health care especially in developing countries [8]. Thus priority setting becomes inevitably value-laden and political, requiring credible evidence and strong and legitimate institutions and fair processes [9–12]. The resource scarcity in developing countries is compounded by the burden of underdevelopment which increases the gap between the health needs and resources available to respond to them; this is in addition to many uncertainties in priority setting due to lack of dependable information [10].

There is need for a programme to generate consensus about a core set of research issues that urgently require attention in order to facilitate policy development. This is known as priority-setting process [12–13]. Campbell [6] identified two major types of priority-setting processes: priority setting for research (determining, weighting and ranking specific research topics and/or research questions) and priority setting for service delivery (determining, weighting and ranking the interventions a health care institution offers - also called #rationing# or intervention priority setting). A balanced process for setting priorities can harmonize competing interests, ground value systems, encourage problem-based learning, resolve conflict, find consensus and ultimately create a set of agreed-upon priorities [12].

The World Health Organization (WHO) has observed that failing or inadequate health systems are one of the main barriers to scaling-up interventions towards the achievement of the millennium development goals [14]. Evidence from a number of reports on health systems strengthening have indicated that there is an urgent need for a stronger body of knowledge about which health policy and health system strengthening strategies are effective, in the context of limited resources [15–17]. Research priority setting is acknowledged to be one of the most vital health system strengthening strategies and a key function of national health research systems which can ensure the alignment of research funding with national evidence needs [18].

In Nigeria as in most low - and middle-income countries (LMICs), there is no rational process in place to set health research priorities especially at State and local government levels [19]. Bryant [10] noted that decision-makers in developing country healthcare institutions lack guidance with regards to priority setting, while Steen and colleagues [20] added that priority setting in developing countries occurs by chance, not by choice. In most LMICs it has been observed that when priority-setting processes do occur, they are typically disease-driven and without a broader, more integrated systems-level perspective (e.g. determining how research might address one or more health-system building blocks) [12]. Furthermore the pattern of research funding is driven by the interests of research funders, who are often external rather than domestic actors, consequently most of the funded health research do not contribute substantially to health policy and health systems strengthening locally. The overriding objectives of this study were: (i). to enhance the knowledge and understanding of policymakers on research priority setting via a training workshop; (ii) to conduct a research priority setting exercise using the essential national health research (ENHR) approach.

## Methods

### Rationale and basis for the research priority exercise

The severe scarcity of resources in most developing countries including Nigeria has made priority setting a very imperative venture [21]. Logical and transparent appeal to determine priorities guide policy makers in their choice of health interventions [22]. The processes of the health research priority setting aim to produce knowledge that will comprehensively benefit the process of health policy development and health systems improvement [23, 24]. This is why priority setting requires transparent approaches and explicit debate about the principles and criteria that are used to make decisions about allocating health care resources [25, 26].

In addition to the challenges associated with severe lack of resources, Nigeria like any other developing country has socio-cultural values and characteristics that may influence the criteria that can be used to set priorities [19]. This explains why a systematic and transparent process of priority setting involving dialogue is important to ensure that the voice and will of the different stakeholders are heard and respected [18]. Although dialogue may not solve a problem or set actual priorities, but it will build the social relationships, trust and interactions critical to knowledge translation in any health system. In its ideal interpretive form, priority setting selects the right people to brainstorm on the right issues to determine what a society's, a system's, or an institution's priorities are [6]. Although there is growing interest in priority setting, there is little consensus on the best way to carry it out [27]. Different approaches have been proposed, ranging from guidelines, checklists and minimum packages to explicit criteria [28]. Because policy makers in the Nigeria context need to make choices taking into account multiple criteria simultaneously, the development of multi-criteria approaches to priority setting becomes imperative; this has been identified as one of the most important issues in the health system research [28, 29].

The Commission on Health Research for Development (COHRED) proposed the ENHR approaches in order to help correct imbalances in global health and development. According to ENHR guideline, countries are required to develop and retain the capacity to set the research priorities, and research and development agencies, funding bodies and other international actors are required to respect these priorities. [5, 30] Priority-setting exercises based on the ENHR approaches have been attempted in some developing countries, including Benin, Commonwealth Caribbean countries, Guinea, Kenya, Nicaragua, The Philippines, South Africa, and Thailand with the countries identifying the need for research on and for health policy [31, 32]. The ENHR approach to priority setting was adopted in this study because it is characterized by the following: (a). inclusiveness; (b). involvement of a broad range of stakeholders, such as researchers, health care providers and of the community; (c). multidisciplinary and cross-sectoral approach; (d). partnership development; (e). participatory and transparent processes; and (f). systematic analyses of health needs, societal and professional expectations. The design in this study was a qualitative cross-sectional survey technique involving a research priority setting meeting using the ENHR approach.

### Ethical consideration

Approval for this study was obtained from the Senate Committee on Research (SCR) of Ebonyi State University, Abakaliki Nigeria. The approval was based on the agreement that participation in the research was voluntary following informed consent; that participants’ anonymity would be maintained; and that every finding would be treated with utmost confidentiality and for the purpose of this research only.

### Selection of participants

An evidence-to-policy meeting was held in December 2010 in Abakaliki the capital of Ebonyi State Nigeria and policymakers were invited to participate. This meeting was a part of a mentorship programme for evidence-informed policymaking organized to enhance the capacity of Nigerian policymakers in which a research priority exercise was conducted. The target participants included the following: health researchers; directors, project/programme managers, and heads of departments in the health ministry; hospital administrators; chief executive officers of health-based civil society groups; leaders of national health-based associations and health directors/managers in uniform services. In Nigeria, these individuals are described as the key actors in the health policymaking process [33–34].

### The research priority setting exercise

A total of 92 policymakers were invited out of which 90(97.8%) attended the meeting. As a part of the priority setting exercise, a 30minutes lecture was delivered entitled: “*Research Priority Setting for Health Policy*” The lecture issues included: Introduction to priority setting; The current state of priority setting in developing countries; Why set research priorities for health policy; Principles of research priority setting for health policy; Essential elements of health policy research priority setting process; The value of public engagement in health policy research priority setting process; and Convening a health policy research priority setting exercise. A consultative group process was used in the priority setting exercise which was inclusive, participatory, interactive and iterative. The principles of putting local concerns first, working towards equity, and linking research to action were emphasized in the priority setting exercise.

During the priority setting exercise participants were classified into six discussion/dialogue groups corresponding to the WHO's health systems building blocks (Group 1-Health workforce; Group 2- Health finance; Group 3- Leadership & Governance; Group 4- Medical Products & Technology; Group 5 - Service Delivery; Group 6- Health Information & Evidence) in line with their job descriptions and organization's operation. For instance participants that are directors of finance in their organization were grouped into the ‘Health Finance’ group. While those who are involved in patient care eg., directors of public health, nursing services, hospital management etc., were grouped into the ‘Service Delivery’ group. A senior policymaker moderated each of the discussion/dialogue groups for the priority setting. The discussion among the various groups followed a deliberative dialogue process. Campbell defined a deliberative dialogue as “*a process of collective and procedural discussion where an inclusive and representative set of stakeholders consider facts from multiple perspectives, converse with one another to think critically about options, and through reasoned argument refine and enlarge their perspectives, opinions and understandings*” [6]. The general theme of the priority setting exercise was". Health Research Priority Setting for Health Systems Improvement in Ebonyi State". Each group was expected to arrive at a consensus about a research theme/topic and identify areas of the research focus with far reaching policy implications within the health systems building block under their consideration.

In line with the ENHR guidelines outlined by Okello and Chongtrakul [5], three basic principles guided the research priority setting exercise and these included: (i). Putting country priorities first; (ii). Working for equity in development; and (iii). Linking research to action for development. The dialogue/deliberations followed a systematic participatory and transparent process that ensured that the voice and will of the different stakeholders are taken into consideration. The dialogue lasted for about 75minutes. The steps taken by each group in the priority setting exercise is outlined in Table 1.

**Table 1:** The guidelines and outline of key considerations/approaches adopted for the research priority setting exercise among policymakers in Ebonyi State Nigeria. (modified from Okello and Chongtrakul 2000)

Priority setting guidelines	Outline of key considerations/approaches
Identification of research areas	Drawing up initial lists of research areas that emerge from situation analysis/deliberations and inputs from various stakeholders.
Criteria for priority setting	Consensus-building to arrive at a provisional list of priority health problems or broad research issues.
Criteria category 1: Appropriateness- (Should we do it?)	Whether the proposed research is well suited to the target society. Ethical and moral issues, human rights issues, legal aspects, political acceptability and commitment of the responsible policy-makers.
Category 2: Relevancy- (Why should we do it?)	Whether the proposed research is the right kind for the right people and that it is pertinent to the health problems of the community, without disregarding equity issues.
Category 3: The chance of success - (Can we do it?)	Capacity of the system to undertake the research, cost justification, time justification and funding support.
Category 4: Impact of the research outcome – (What will the stakeholders get out of it?)	Benefit of using or implementing the research results, and evaluate the merit and usefulness of the research outcome. Research utilisation, public health significance, economic impact and development impact.

## Results

A total of six groups corresponding to WHO′s health systems “building blocks” (service delivery; information and evidence; medical products and technologies; health workforce; health finance; leadership and governance) of 11-16 participants per group participated in the research priority setting exercise. The research themes and the areas of research focus identified by the participants are presented in Table 2. It was the consensus of the policymakers of the Medical products and Technology group that research should focus on Procurement practices, quality assurance, storage system and challenges of optimal access to health products and technology in Nigeria. According to them, emphasis should be placed on strengths and weaknesses of existing procurement practices, issues and frameworks for quality assessment/assurance and inventory management systems. Their counterparts of the Health Service Delivery were of the consensus that research should focus on effective health service delivery, disease prevention and control under a national emergency situation. They were of the opinion that research emphasis should be placed on the framework for managing national/state health emergency situations (eg., disease outbreaks), health education, immunization services, treatment/control of locally endemic diseases and resource mobilization and allocation under emergencies. The Health workforce group was of the consensus that research should be directed towards addressing the shortfalls in the supply of professional personnel and labour crisis in the Nigerian health sector. While the Leadership and Governance group emphasized the need for research to be focused on addressing the issues of governance in the health sector management in Nigeria.

**Table 2:** The research themes and identified areas of research focus from the priority exercise adopted by policymakers in Ebonyi State Nigeria

Health systems group	Research theme selected	Research priority areas of focus identified
**1. Health Information & Evidence**	Grassroots health data generation and management in Nigeria: Challenges and strategies.	(i). Framework for systematic data collection at different levels of health operations. (ii). Managing and interpreting grassroots health data. (iii). Transforming grassroots health data to national/state health data.
**2. Medical Products & Technology**	Procurement practices, quality assurance, storage system and challenges of optimal access to health products and technology in Nigeria.	(i). Strengths and weaknesses of existing procurement practices. (ii). Issues and frameworks for quality assessment/assurance (iii). Inventory management systems.
**3. Health Financing**	Financial resource mobilization, utilization and sustainable health system management in Nigeria.	(i). Available funding sources at different levels of governance eg., National, State, Local government levels. (ii). Budgetary provisions for the health sector. (iii). Cash flow management.
**4. Health Workforce**	Shortfalls in the supply of professional personnel, remuneration matters and labour crisis in the Nigerian health sector.	(i). Shortages of health workers. (ii). Persistent demand for increases in wages and salaries. (iii). Incessant strikes and picketing among health workers.
**5. Health Service Delivery**	Effective health service delivery, disease prevention and control under a national emergency situation.	(i). Framework for managing national/state health emergency situations (eg., disease outbreaks). (ii). Health education/ Immunization services (iii). Treatment/control of locally endemic diseases.
**6. Leadership and Governance**	Governance issues in health sector management in Nigeria.	(i). Health policy formulation and implementation. (ii). Legal and professional environment for health services management. (iii). Structures for effective health systems management.

## Discussion

The importance of research priority setting cannot be overstated because priority setting is arguably most important when resources are scarce, as is the case in Nigeria. In spite of the huge importance of research priority setting to the health policymaking process, available reports indicate that policymakers in developing countries are rarely involved in it [9, 35]. In the present project the research priority setting exercise conducted was the first experience to over 85% of the participating policymakers. Ranson and Bennett [17] noted in their report that priority setting for health research is often not performed well - or not performed at all. Furthermore in a survey of more than 550 policy makers and almost 1, 900 researchers in 13 low- and middle-income countries, it was observed that about a third of policy-makers, researchers and users of research interviewed said that there was either no rational process to set health research priorities in their country or that they were unaware of how priorities were identified or set [35].

Research priority setting exercise was introduced in this study in order to sensitize the policymakers and other participating stakeholders of its value as an example of demand driven strategy in the health policymaking process capable of reversing inequities and strengthening the health systems. Although there is currently insufficient evidence that the use of priority-setting strategy improves health outcomes and reverses existing inequities [4], it is pertinent to state however that evidence abound indicating that the lack of a rational and transparent process such as research priority setting generates inequity and stagnation in mortality levels [36–38]. It has been argued that priority setting processes should be demand driven, and involve multiple different types of informational inputs as well as multiple stakeholder perspectives [18]. Lomas and colleagues also noted that research priority setting exercise is an important step in the ongoing linkage and exchange between those who fund and conduct applied health services research and the stakeholders whom the research aims to influence [39].

The involvement of multiple categories of stakeholders of the health sector in the priority setting exercise conducted in this study was an outstanding accomplishment because it not only afforded the participants the opportunity to establish linkage and exchange but also enabled inputs to be made from wider representatives of the society. This approach is undoubtedly a paradigm shift in the way in which research is usually produced and consumed. Hunter [40] noted that rather than academics exclusively setting the research agenda, a new approach to knowledge co-creation is overdue whereby researchers, and those they are seeking to address, work together to define the research questions, agree the methods, and assess the implications of the data analysis and findings for policy and practice. The participation of a broadened spectrum of stakeholders helps to identify research needs, technical and financial capabilities, information gaps and distortions, the political environment, and the values and ethics of a given society [18]. According to Ranson and Bennett [17], not bringing certain groups - such as policy makers and civil society organizations into the priority setting process may contribute to the neglect of certain health research fields, including health policy and systems research (HPSR). Involving major stakeholders in priority setting fosters ownership of both process and output, and facilitates shared responsibility and accountability in the implementation of the research agenda [18].

In the priority setting exercise conducted in this study the consultative group process was used. Although the consultative group process is systematic, it nevertheless ensures that opinion and will of the different stakeholders are heard and respected [5, 18]. Each of the dialogue groups corresponding to WHO's health systems building block selected a research topic from among identified priority problems along with specific areas of focus for the research following a deliberative dialogue process. Deliberative dialogue has been described as a very useful mechanism of enhancing the quality of research priority setting because it adds both scientific and social credibility to the decision-making process, as it unites and empowers those who will be affected by the eventual decision [41].

Interestingly, the core principles of putting local/country concerns first and linking research to action were emphasized as the basis for the priority setting exercise. This explains why the research themes/topics selected by the policymakers and other participating stakeholders in the priority setting exercise focused much more on specific local context health challenges. For instance the health information and evidence group selected the topic: *‘Grassroots health data generation and management in Nigeria: Challenges and strategies’*, while their counterparts of the medical products and technology group selected the topic: *‘Procurement practices, quality assurance, storage system and challenges of optimal access to health products and technology in Nigeria’*. This approach is in line with the COHRED concept of ENHR recognized as a strategy for promoting health and development on the basis of equity and social justice at national and sub-national levels [5, 13].

The outcome of this study suggests that a research priority exercise can be successfully conducted among policymakers in a low income setting. This is an important step towards evidence-informed policymaking and practice. However, it is not enough to set research priorities. There must be mechanism in place to ensure the implementation of the research priorities. Okello and Chongtrakul[5] outlined seven steps towards the implementation of research priorities as follows: (i). Building and facilitating interdisciplinary and multi-stakeholder teams; (ii). Identification of resources by priority area; (iii). Research protocol development; (iv). Establishment of a peer-review process and a forum for revision; (v). A mechanism for monitoring and evaluation of research work; (vi). Dissemination of research findings; and (vii). Utilization of research results.

## Conclusion

Priority setting is not a one-time activity but needs to be done periodically as priorities keep changing based on the changing health care demands and the availability of resources. It is important to establish bodies or structures with some powers to influence the policies, within the government set up or in close proximity to the government set up, which are given the mandate of doing periodic priority setting exercise. It is of utmost importance to include appropriate level of policy makers in such bodies, so that the priority setting can influence the policies and resource allocation. Such bodies can review the set priorities based on what is implemented, the new demands and the resources available. These bodies can also follow over a longer period of time if priority setting has any impact on policy. The research priority setting process adopted in this study as well as the implementation steps outlined by Okello and Chongtrakul [5] are recommended for LMIC settings similar to the Nigeria situation.
